# Supplementary material for: Distinct evolution of SARS-CoV-2 Omicron XBB and BA.2.86/JN.1 lineages combining increased fitness and antibody evasion
Source: Nat Commun. 2024 Mar 13;15:2254. doi: 10.1038/s41467-024-46490-7 (PMC10938001; doi:10.1038/s41467-024-46490-7)
Supplement: Supplementary file 1 — Supplementary Information [file 41467_2024_46490_MOESM1_ESM.pdf]

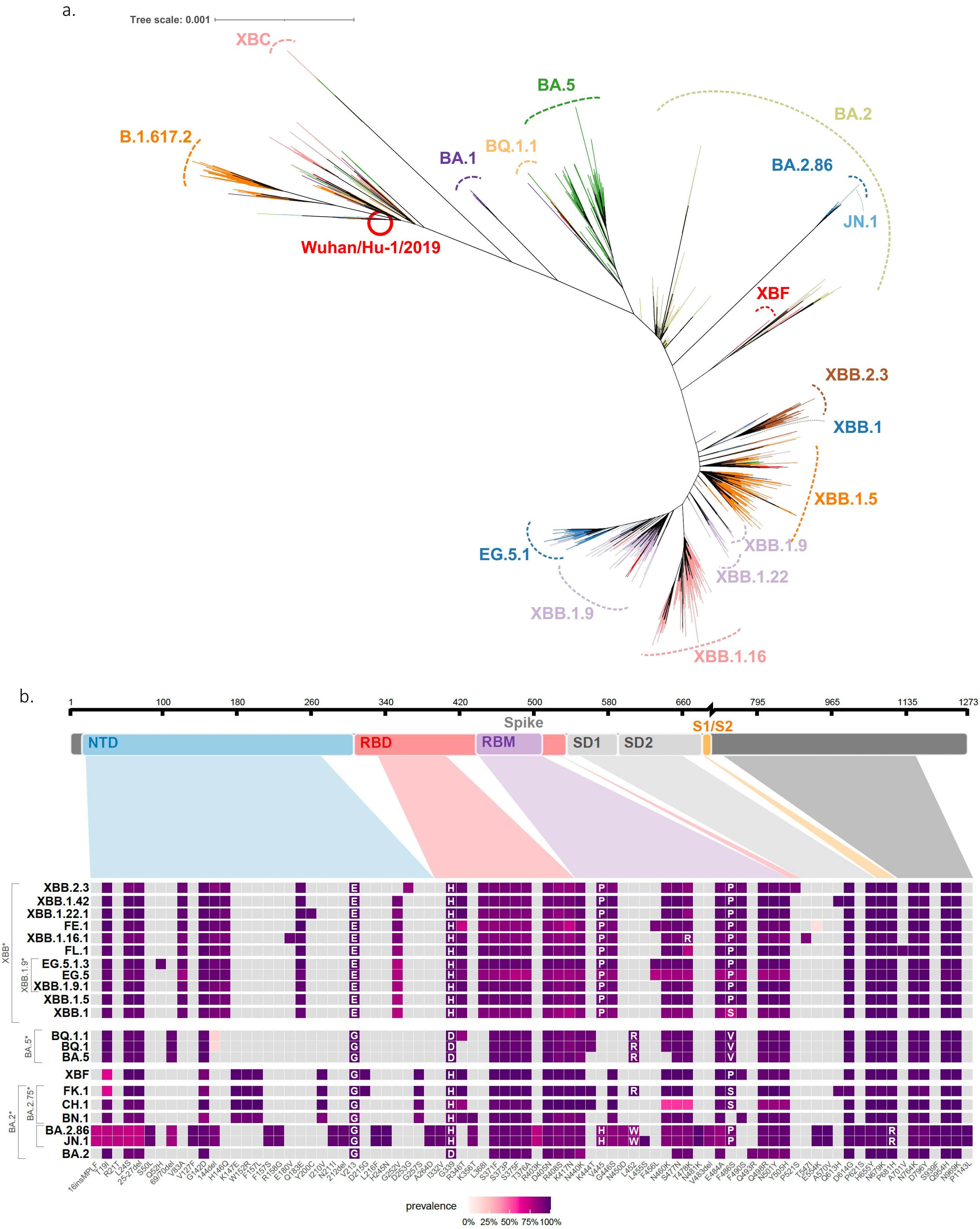

Figure S1. Maximum likelihood phylogeny representing the evolution of the main lineages of SARS-CoV-2 since the beginning of the epidemic. **a.** The red circle indicates one of the first sequences (Wuhan/Hu-1/2019). The BA.2.86 sequences are located inside the BA.2 clade, at the end of a long branch. Of note, a limitation of the inferred phylogeny is that known recombinant lineages (e.g. XBF or XBB) were included in the dataset, and their branching should be interpreted with caution. **b.** Comparison of spike mutations compared to the reference Wuhan_Hu-1 for selected SARS-CoV-2 variants. The color scale reflects the frequency of the mutations within lineages based on the data available on the GISAID EpiCoV database.

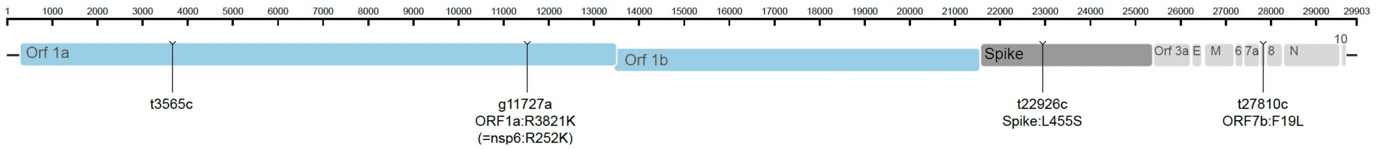

Figure S2. Additional mutations defining lineage JN.1 in comparison to its parent lineage BA.2.86.1. The genome is drawn to scale, and for mutation leading to an amino acid change, the protein is specified.

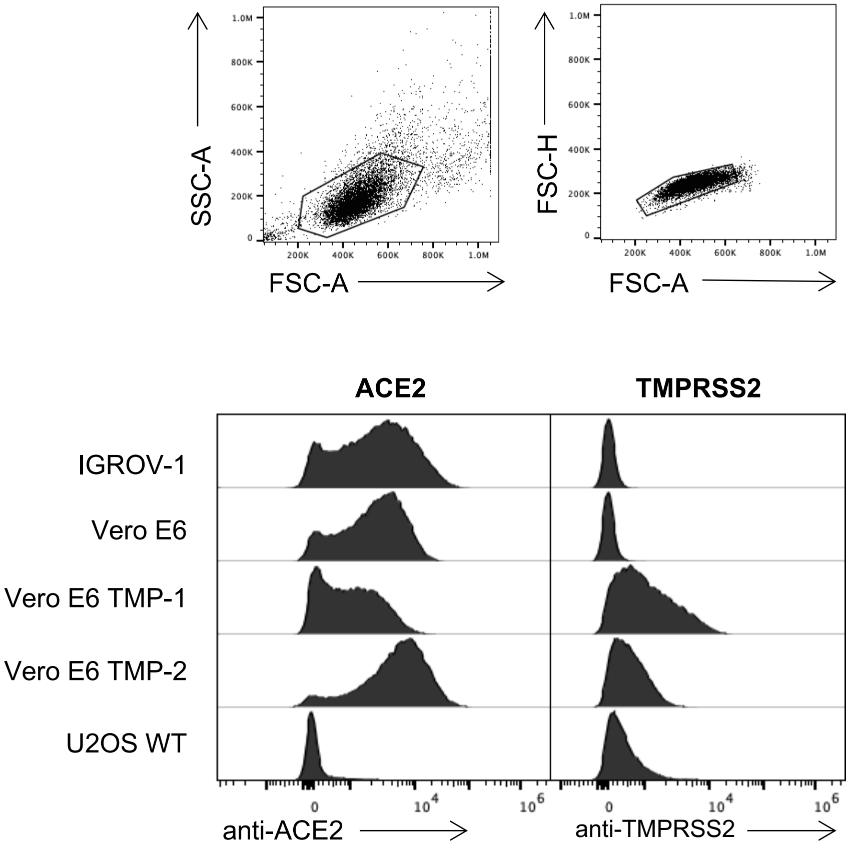

Figure S3. ACE2 and TMPRSS2 expression in cell lines. IGROV-1, Vero E6, Vero E6 TMP-1 and TMP-2 cells were stained with anti-ACE2 or anti-TMPRSS2 antibodies and analyzed by flow cytometry. Representative examples of the gating strategy in IGROV-1 cells and of the signals obtained in cell lines from n=3 independent experiments are shown.

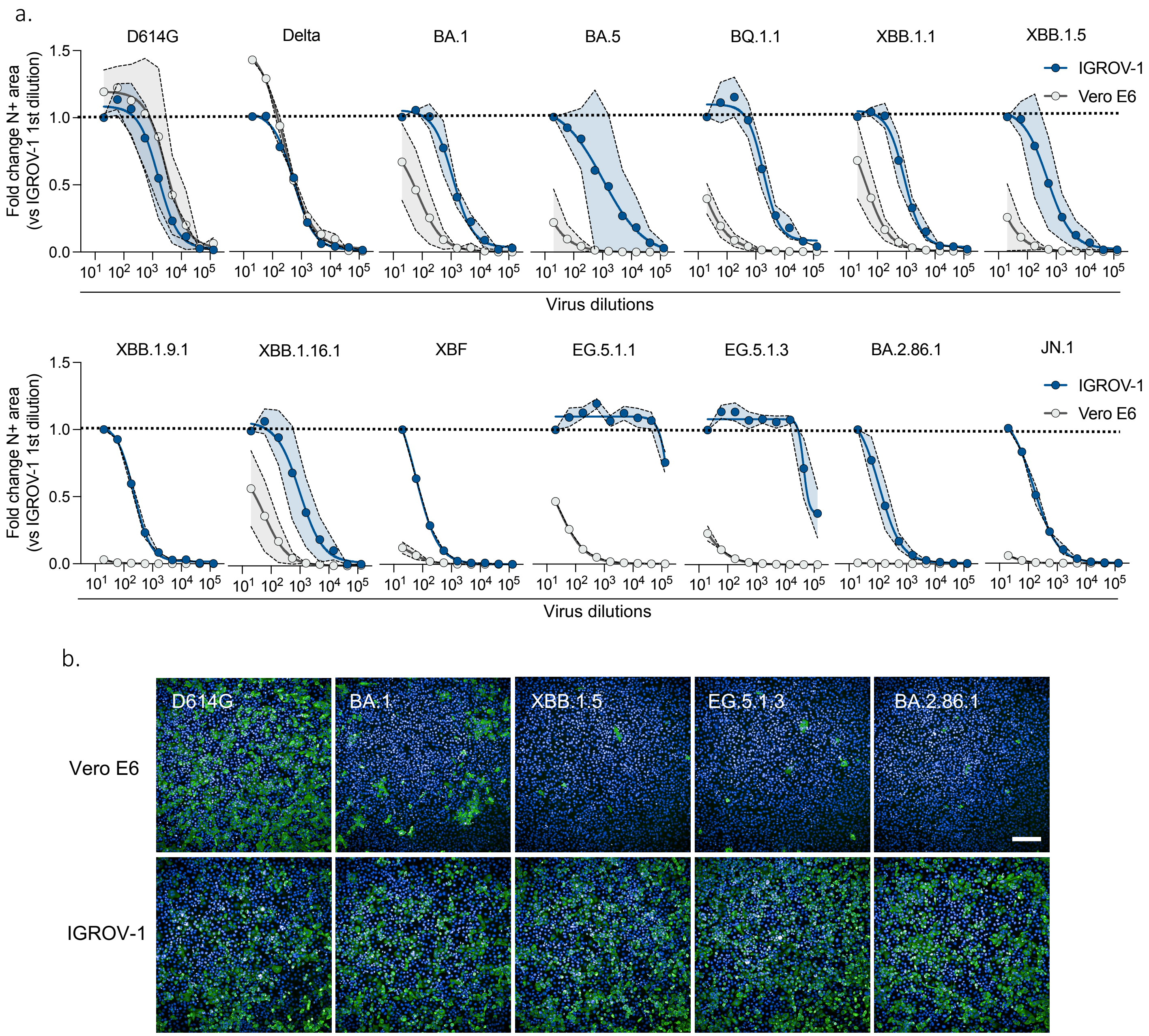

Figure S4. Permissibility of IGROV-1 and Vero E6 cells to SARS-CoV-2 variants. Viral titrations in IGROV-1 and Vero E6 cells. Cells were infected with serial dilutions of the indicated variants. At 24 hours post-infection (p.i.), cells were stained with a pan-coronavirus anti-N antibody (green) and Hoechst to visualize the nuclei (blue). **a.** The N positive areas in IGROV-1 (blue curves) and Vero E6 cells (grey curves) were compared for each viral dilution. The dotted line corresponds to the signal obtained with the first dilution (1/20) in IGROV-1 cells, set to the arbitrary value of 1. Data are mean ± s.d. of 5 independent experiments (n=2 for JN.1). **b.** Representative images with the indicated viral strains. An identical viral inoculum was used in the two cell lines. Scale bar, 200 µm.

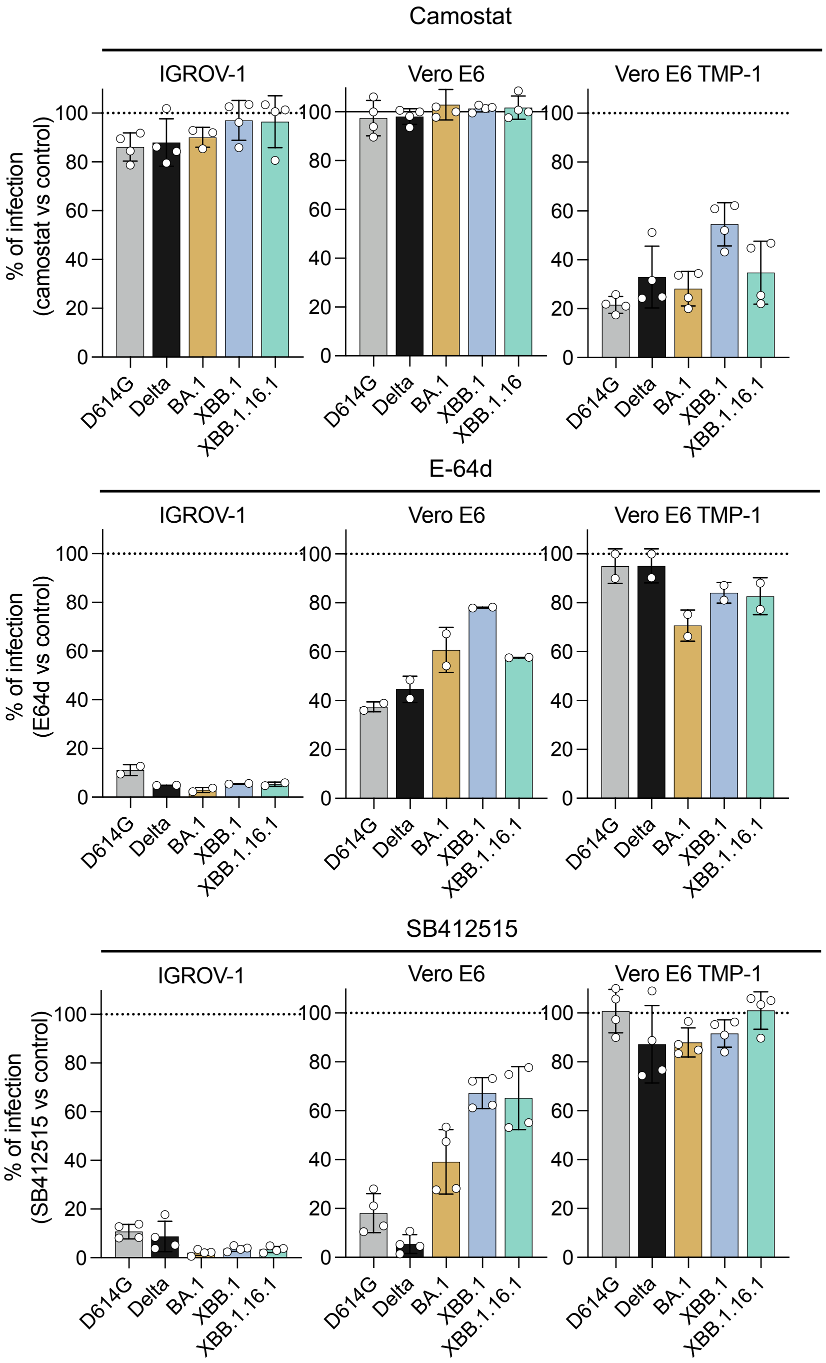

Figure S5. Effect of the TMPRSS2 inhibitor Camostat and Cathepsin L inhibitors E-64d and SB412515 on viral infection IGROV-1, Vero E6 and Vero E6 TMP-1 cells pre-incubated 2 h with Camostat (100 µM), E-64d (10 µM), or SB412515 (10 µM) and exposed to the D614G, Delta, BA.1, XBB.1, or XBB.1.16.1 variants for 24 h. The viral inoculum was determined by titration in each cell line to achieve a similar N-positive area at 24 h pi. The cells were stained with a pan-coronavirus anti-N antibody. Hoechst was used to visualize the nuclei for evaluating drug toxicity. The percentage of infection compared to the control (without drugs, represented by the dotted line) is shown. Data are mean ± s.d. of 4 independent experiments (2 independent experiments for E-64d).

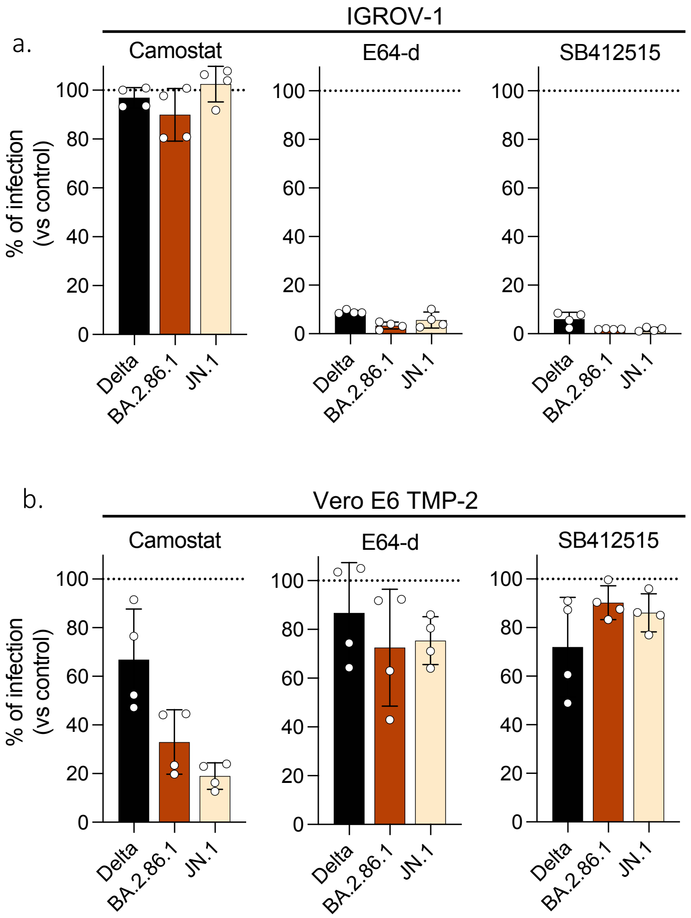

Figure S6. Sensibility of BA.2.86.1 and JN.1 variants to TMPRSS2 inhibitor Camostat and Cathepsin L inhibitors E-64d and SB412515. IGROV-1 **(a)** and Vero E6 TMP-2 **(b)** cells pre-incubated 2 h with Camostat (100 µM), E-64d (10 µM), or SB412515 (10 µM) and exposed to the Delta, BA.2.86.1 and JN.1 variants for 24 h. The viral inoculum was determined by titration in each cell line to achieve a similar N-positive area at 24 h pi. The cells were stained with a pan-coronavirus anti-N antibody. Hoechst was used to visualize the nuclei for evaluating drug toxicity. The percentage of infection compared to the control (without drugs, represented by the dotted line) is shown. Data are mean ± s.d. of 2 independent experiments.

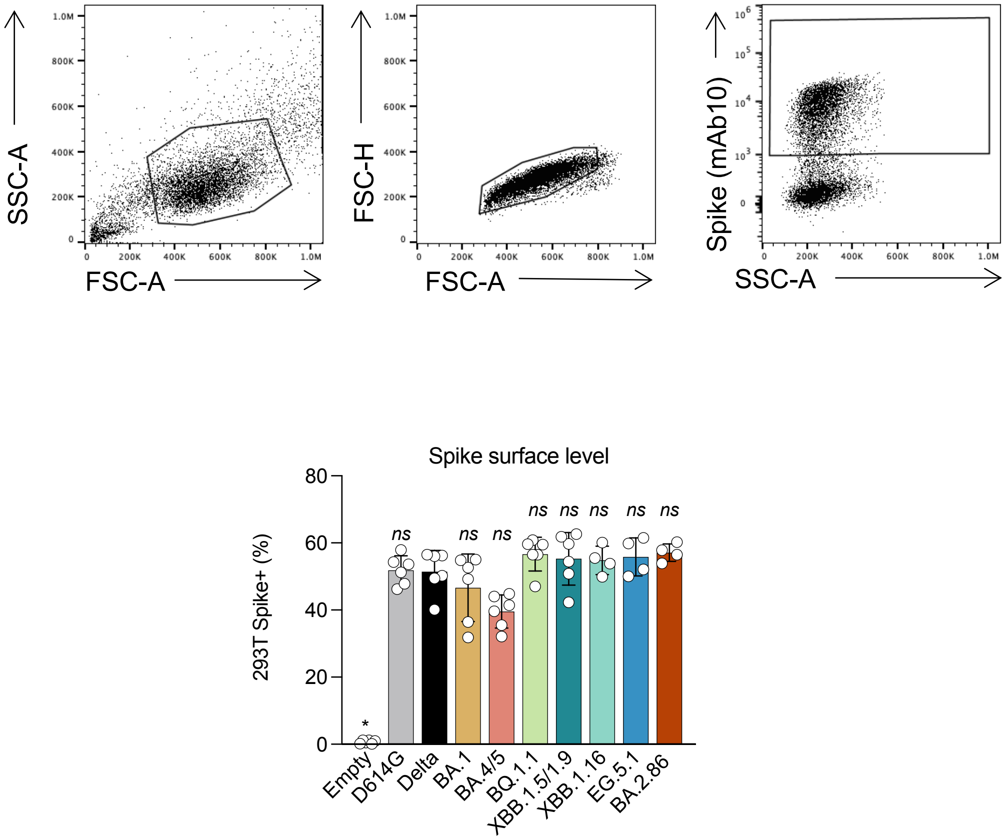

Figure S7. Spike expression in 293T cells. Gating strategy and levels of Spike at the cell surface. 293T cells transiently expressing the indicated variant Spikes were stained with a pan-coronavirus anti-S2 mAb 18 h post-transfection and analyzed by flow cytometry. Data are mean ± s.d. of 6 independent experiments (n=4 for XBB.1.16.1, EG.5.1.1 and BA.2.86.1). One-way ANOVA with Kruskal-Wallis test followed by Dunn's test for multiple comparisons to compare Delta with respective variants were conducted.

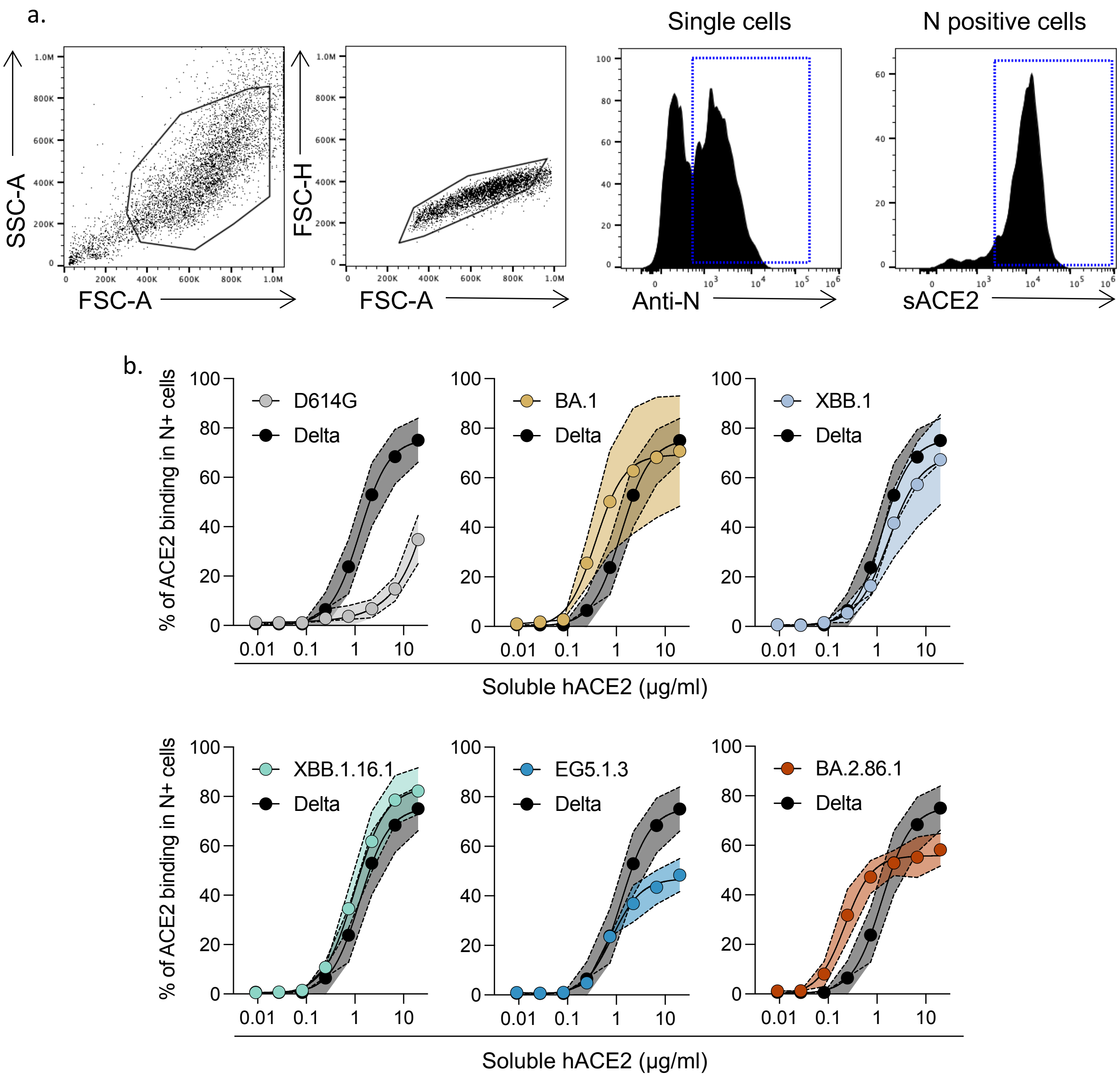

Figure S8. Binding of variant Spike proteins to ACE2. The affinity of the variant Spike proteins expressed at the surface of IGROV-1 infected cells was assessed by staining the cells and flow cytometry analysis. IGROV-1 cells were infected with the indicated variants. a. Representative examples of the gating strategy. b. The percentages of biotinylated hACE2+ cells are represented at various dilutions of hACE2-Fc. Data are mean ± s.d. of 3 independent experiments.

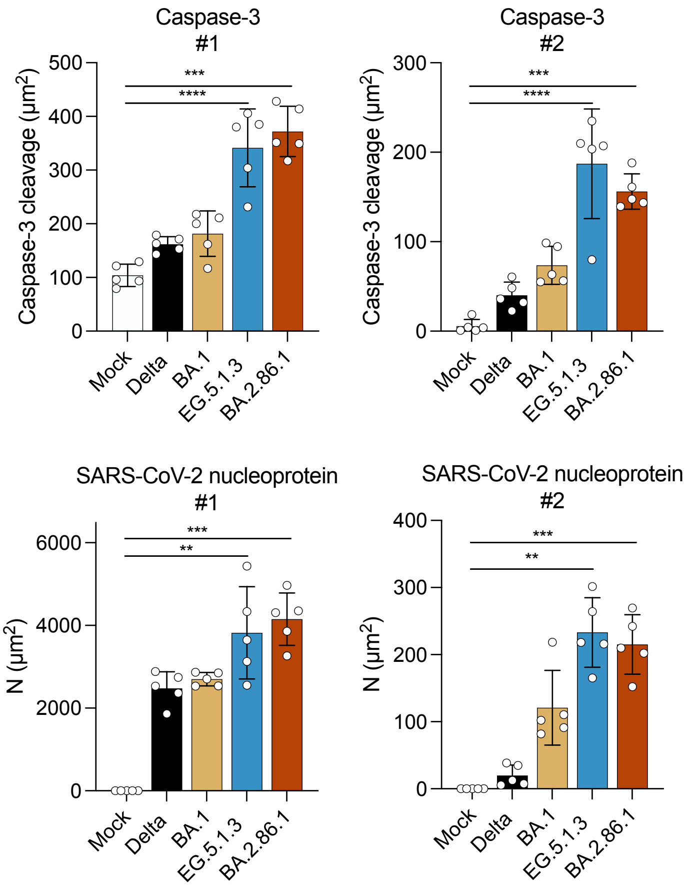

Figure S9. Immunofluorescence of hNECs stained for cleavage products of caspase-3 and SARS-CoV-2 nucleoprotein. Quantification of total area of cleavage products of caspase-3 and SARS-CoV-2 N. Each data point represents one randomly assigned field. One-way ANOVA with Kruskal-Wallis test followed by Dunn's test for multiple comparisons to compare Mock with respective variants were conducted

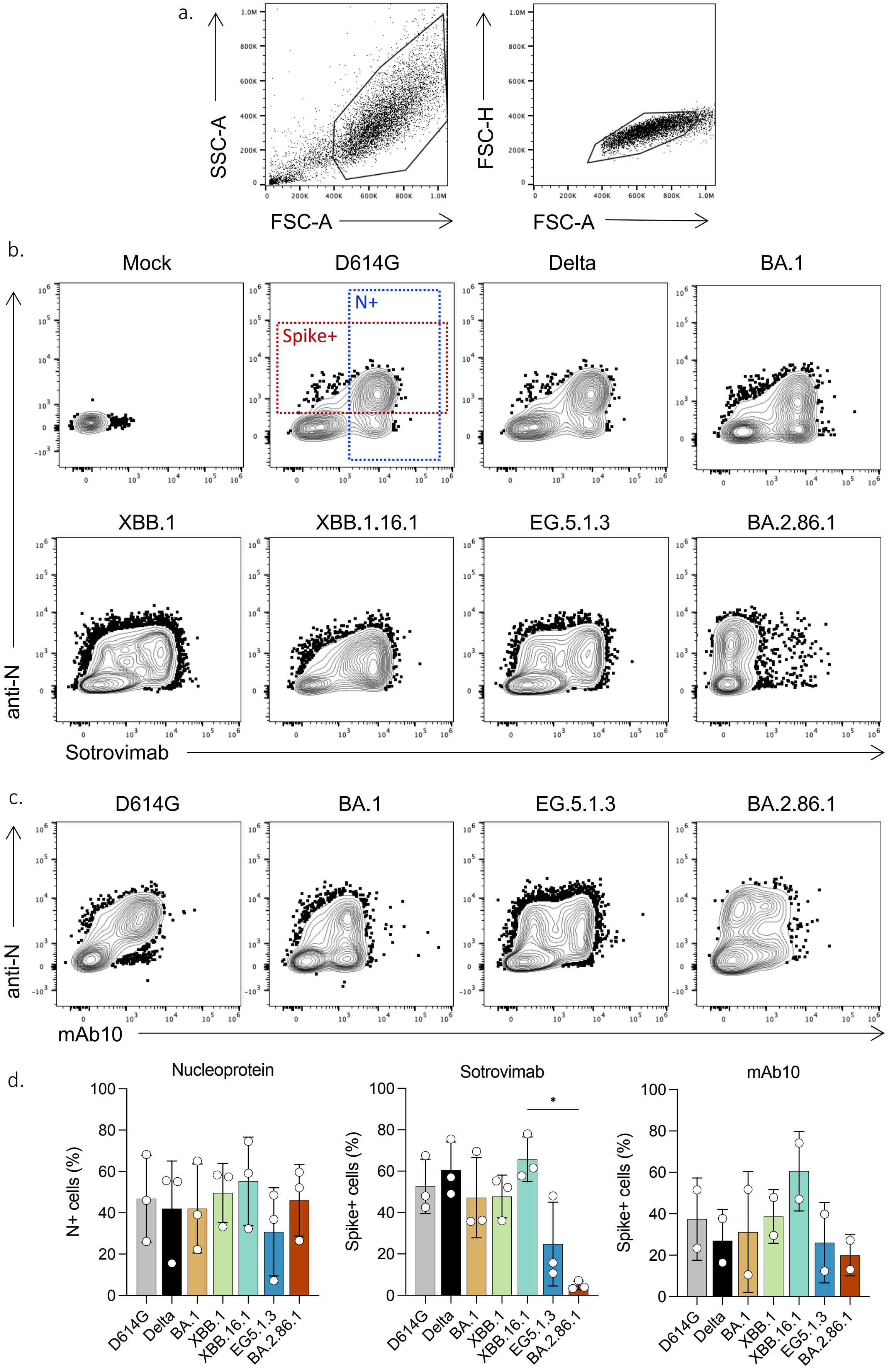

Figure S10. Sotrovimab binding to infected cells. IGROV-1 cells were infected with the indicated virus for 24h. Then, cells were stained with Sotrovimab or the pan-coronavirus anti-S2 mAb10 antibody. After fixation, cells were intracellularly stained with anti-SARS-CoV-2 nucleoprotein (N) antibody NCP-1 and analyzed by flow cytometry. Representative examples of the gating strategy **(a)** and co-staining of Nucleocapsid and Sotrovimab **(b)** or mAb10 **(c)**. **d.** Quantification of the SARS-CoV-2 nucleoprotein+ cells (left, n=3), Spike+ cells strained with Sotrovimab (middle, n=3) and mAb10 (right, n=2). Gating strategy for N+ cells (blue gate) and S+ cells (red gate) is shown in S10b. One-way ANOVA with Kruskal-Wallis test followed by Dunn's test for multiple comparisons were conducted.

a. **Orléans Cohort: Individuals vaccinated with bivalent Original/BA.5 mRNA Pfizer**

Patients ID	Age	Sex	Dose 3 (Pfizer Monovalent)	Dose 4 (Pfizer Monovalent)	Dose 4 or 5 (Pfizer bivalent)	Breakthrough	Days post-bivalent boost			
							before	1M	3M	6M
#1	50-59	M	Jan-22		Dec-22		0	30	/	/
#2	50-59	M	Aug-21	Jan-22	Dec-22		0	35	/	/
#3	50-59	M	Aug-21	Jan-22	Dec-22	Apr-22	0	42	/	/
#4	Unknown	M	Nov-21		Dec-22	Yes (date unknown)	0	53	/	/
#5	60-69	M	Nov-21		Dec-22	Mar-22	0	26	96	/
#6	50-59	F	Nov-21		Dec-22	Mar-22	0	36	97	/
#7	30-39	F	Feb-22		Dec-22	Jan-21	0	37	93	/
#8	60-69	M	Dec-21		Dec-22		0	45	94	/
#9	40-49	F	Nov-21		Dec-22	Mar-22	0	35	93	/
#10	40-49	F	Nov-22		Dec-22	Mar-22	0	28	91	/
#11	30-39	F	Nov-21		Dec-22	Mar-22	0	27	84	/
#12	40-49	F	NA		Dec-22	Jan-22	0	29	/	/
#13	60-69	F	Nov-21	Jun-21	Jan-23		0	/	/	/
#14	60-69	F	Jun-21	Aug-22	Feb-23	Dec-21	/	/	/	128
#15	70-79	M	Aug-21	Aug-22	Feb-23	Dec-21	/	/	/	128
#16	50-59	M	Aug-21		Feb-22		/	/	/	484
#17	60-69	M	Feb-22		Dec-22	Aug-21	/	/	/	195
#18	60-69	M	Sep-21		Feb-23	Jan-22	/	/	/	154
#19	50-59	M	Aug-21	Jan-22	Dec-22		/	/	/	202
#20	70-79	M	Sep-21		Dec-22	Aug-22	/	/	/	216
#21	60-69	M	Aug-21	Jan-22	Dec-22	Feb-22	/	/	/	202

b. **Lyon Cohort_Vaccinated with bivalent Original/BA.5 Pfizer**

Patients ID	Age	Sex	Dose 3 (Pfizer Monovalent)	Dose 4 (Pfizer Monovalent)	Dose 4 or 5 (Pfizer bivalent)	Breakthrough	Days post-bivalent boost		
							before	1M	6M
#1	50-59	F	Nov-21		Dec-22	Jun-22	0	32	207
#2	30-39	M	Nov-21	Jan-22	Dec-22	Jul-22	0	31	217
#3	60-69	F	Dec-21	Jan-22	Dec-22	/	0	28	166
#4	40-49	F	Dec-21		Dec-22	Feb-22	0	32	218
#5	60-69	F	Oct-21		Dec-22	/	0	30	136
#6	60-69	F	Nov-21		Dec-22	/	0	28	165
#7	30-39	M	Nov-21		Dec-22	Jan-22	0	38	152
#8	60-69	F	Nov-21		Dec-22	/	0	32	/

c. **Orléans Cohort_Breakthrough infection in September 2023**

Patients ID	Age	Sex	Number of Doses	Vaccine	Days post-infection
#1		M	3	Pfizer	50
#2		M	3	Pfizer	42
#3		F	3	Pfizer	14
#4		F	2	Pfizer	39
#5		F	2	Pfizer	24
#6		F	3	Pfizer	20
#7		M	3	Pfizer	10
#8		F	3	Pfizer	25
#9		F	3	Astra + Pfizer	15
#10		M	2	Pfizer	18
#11		F	3	Pfizer	17
#12		F	2	Pfizer + Moderna	14

Table S1

a.

Post third dose		
Sex	Female	13
	Male	8
Age (Median; range)		57; (31-69)
Immunodeficiency		0
Previous COVID-19 (known)		13
1st dose		jan - april 2021
2nd dose		jan - nov 2021
3rd dose		aug 2021 - nov 2022
4th dose		june 2021 - jan 2022
Sampling days post-vaccination (median; range)		373 (25-475)
Cohorts	Orléans (Table S1a)	13
	Lyon (Table S1b)	8

b.

1M post-bivalent (Original/BA.5) dose		
Sex	Female	12
	Male	8
Age (Median; range)		56; (31-69)
Immunodeficiency		0
Previous COVID-19 (known)		13
1st dose		jan - april 2021
2nd dose		jan - nov 2021
3rd dose		aug 2021 -nov 2022
4th dose		nov 2022 - jan 2023
Sampling days post-bivalent boost (median; range)		32 (26-53)
Cohorts	Orléans (Table S1a)	12
	Lyon (Table S1b)	8

c.

6M post-bivalent (Original/BA.5) dose		
Sex	Female	6
	Male	9
Age (Median; range)		62; (34-76)
Immunodeficiency		0
Previous COVID-19 (known)		9
1st dose		janv-21
2nd dose		janv-21
3rd dose		june 2021 - feb 2022
4th dose		feb 2022 - feb 2023
Sampling days post-bivalent boost (median; range)		198 (128-484)
Cohorts	Orléans (Table S1a)	8
	Lyon (Table S1b)	7

d.

0.5M post XBB breakthrough infection		
Sex	Female	8
	Male	4
Age (Median; range)		Unknown
Immunodeficiency		Unknown
Nb of participant with 2 doses		4
Nb of participant with 3 doses		8
Sampling days post-BT (median; range)		19 (10-50)
Cohorts	Orléans (Table S1c)	12

Table S2

Variant	Strain	GISAID ID	Reference
D614G	hCoV-19/France/GES-1973/2020	EPI_ISL_414631	Ref 68
Delta	hCoV-19/France/IDF-APHP-HEGP-20-23-2131905084/2021	EPI_ISL_2029113	Ref 65
BA.1	hCoV-19/Belgium/rega-20174/2021	EPI_ISL_6794907	Ref 52
BA.5	hCoV-19/France/CVL-ChuTo-9800086928/2022	EPI_ISL_13660702	Ref 53
BQ.1.1	hCoV-19/Belgium/Rega-IPP_BA2752i/2022	EPI_ISL_15731524	Ref 34
XBB.1.5	hCoV-19/USA/NY-PRL-2021_0412_01L21/2021	EPI_ISL_1635849	Ref 48
XBB.1	hCoV-19/France/PAC-HCL022171892001/2022	EPI_ISL_15619797	This article
XBB.1.9.1	hCoV-19/France/GES-IPP08594/2023 C2 24/04/2023	EPI_ISL_17419152	This article
XBB.1.16.1	hCoV-19/France/GES-IPP07712/2023 C2 21/04/2023	EPI_ISL_17383796	This article
XBF	hCoV-19/France/IDF-APHP-HEGP-81-10-2332993394/2023	EPI_ISL_17763463	This article
EG.5.1.1	hCoV-09/France/GES-IPP15954/2023	EPI_ISL_17949406	This article
EG.5.1.3	hCoV-19/France/BRE-IPP15906/2023	EPI_ISL_17949372	This article
BA.2.86.1	hCoV-19/France/IDF-IPP17625/2023	EPI_ISL_18221650	This article
JN.1	hCoV-19/France/HDF-IPP21391/2023	EPI_ISL_18363371	This article

Table S3

	Fold decrease versus D614G										
	**BA.1**	**BA.5**	**BQ.1.1**	**XBB.1**	**XBB.1.5**	**XBB.1.9.1**	**XBB.1.16.1**	**XBF**	**EG.5.1.3**	**BA.2.86.1**	**JN.1**
3 doses of Pfizer	5	5	23	42	35	84	101	64	136	76	101
1M after Original/BA.5 bivalent boost	9	6	50	107	62	150	144	71	141	125	254
6M after Original/BA.5 bivalent boost	4	7	23	31	37	53	75	37	81	90	180
3 weeks after XBB breakthrough infection	2	1	6	8	4	8	9	5	6	10	20

Table S4
