# Supplementary material for: Distinct evolution of SARS-CoV-2 Omicron XBB and BA.2.86/JN.1 lineages combining increased fitness and antibody evasion
Source: Nat Commun. 2024 Mar 13;15:2254. doi: 10.1038/s41467-024-46490-7 (PMC10938001; doi:10.1038/s41467-024-46490-7)
Supplement: Supplementary file 3 — Reporting Summary [file 41467_2024_46490_MOESM3_ESM.pdf]

nature portfolio

Corresponding author(s): Olivier Schwartz
Etienne Simon-Loriere
Delphine Planas

Last updated by author(s): Feb 28, 2024

Reporting Summary

Nature Portfolio wishes to improve the reproducibility of the work that we publish. This form provides structure for consistency and transparency in reporting. For further information on Nature Portfolio policies, see our Editorial Policies and the Editorial Policy Checklist.

Statistics

For all statistical analyses, confirm that the following items are present in the figure legend, table legend, main text, or Methods section.

n/a	Confirmed	
☐	☒	The exact sample size (*n*) for each experimental group/condition, given as a discrete number and unit of measurement
☐	☒	A statement on whether measurements were taken from distinct samples or whether the same sample was measured repeatedly
☐	☒	The statistical test(s) used AND whether they are one- or two-sided<br>*Only common tests should be described solely by name; describe more complex techniques in the Methods section.*
☐	☒	A description of all covariates tested
☐	☒	A description of any assumptions or corrections, such as tests of normality and adjustment for multiple comparisons
☐	☒	A full description of the statistical parameters including central tendency (e.g. means) or other basic estimates (e.g. regression coefficient) AND variation (e.g. standard deviation) or associated estimates of uncertainty (e.g. confidence intervals)
☐	☒	For null hypothesis testing, the test statistic (e.g. *F*, *t*, *r*) with confidence intervals, effect sizes, degrees of freedom and *P* value noted<br>*Give P values as exact values whenever suitable.*
☒	☐	For Bayesian analysis, information on the choice of priors and Markov chain Monte Carlo settings
☒	☐	For hierarchical and complex designs, identification of the appropriate level for tests and full reporting of outcomes
☒	☐	Estimates of effect sizes (e.g. Cohen's *d*, Pearson's *r*), indicating how they were calculated

Our web collection on statistics for biologists contains articles on many of the points above.

Software and code

Policy information about availability of computer code

Data collection	Harmony Software v4.9 (Perkin-Elmer), Attune Nxt Software v3.2.1 (ThermoFischer), Flowjo Software vlü.7.1, GISAID Epicov database (https://gisaid.org/)
Data analysis	Excel 365 vl6.46 (Microsoft), Prism v9.0.2 (Graph Pad Software), Trimmomatic v0.39, mega hit vl.2.9, CLC Assembly Cell vS.1.0,G eneious prime v2023.2.l, DIAMOND v2.0.4,p angolin v4.3.l, nextalign v2.14.0,g ofasta vl.2.1, goalign v0.3.5, iqtree v2.2.0, gotree v0.4.4, R v4.3, ggplot 3.4.3. The bioinformatic workflow used is available on Github https://github.com/SimonLoriereLab/sarscov2_Oct2023 , and has been deposited on Zenodo (https://zenodo.org/doi/10.5281/zenodo.10692772).

For manuscripts utilizing custom algorithms or software that are central to the research but not yet described in published literature, software must be made available to editors and reviewers. We strongly encourage code deposition in a community repository (e.g. GitHub). See the Nature Portfolio guidelines for submitting code & software for further information.

Data

Policy information about availability of data

All manuscripts must include a data availability statement. This statement should provide the following information, where applicable:

- Accession codes, unique identifiers, or web links for publicly available datasets
- A description of any restrictions on data availability
- For clinical datasets or third party data, please ensure that the statement adheres to our policy

All data supporting the findings of this study are available within the article or from the corresponding authors upon reasonable request without any restrictions.

The sequences of the viral stocks of the viruses isolated in this study have been deposited on GenBank (Accession numbers: PP405601-PP405606).

Research involving human participants, their data, or biological material

Policy information about studies with human participants or human data. See also policy information about sex, gender (identity/presentation), and sexual orientation and race, ethnicity and racism.

Reporting on sex and gender	Gender was collected based on self-reporting. Sex or gender analysis was not performed due to the limited number of participants.
Reporting on race, ethnicity, or other socially relevant groupings	Given the exploratory design of the study, race, ethnicity and socially grouping were not pre-established and not reported.
Population characteristics	Given the exploratory design of the study, the characteristics of participants were not pre-established when entering the cohorts. Relevant covariates (age, sex, disease, vaccinations and previous COVID-19) are provided in the corresponding supplementary tables. Nasopharyngeal swabs used for viral isolation were leftover samples from usual care their use for research purposes was authorized by the ethics committee "Comite d'ethique de la recherche AP-HP Centre" affiliated to the AP-HP (Assistance publique des Hopitaux de Paris; IRB registration# 00011928) or provided by the the National Reference Centre for Respiratory Viruses hosted by Institut Pasteur. All Participants or their legal authorized representatives provided a written informed consent.
Recruitment	Individuals were recruted during their visit at the hospitals.<br>Individuals were included without any selection other than those imposed by the entry criteria. Under these conditions, no particular bias is envisaged.
Ethics oversight	The "Orleans" cohort is an ongoing prospective, monocentric, longitudinal, observational cohort clinical study aiming to describe the kinetic of neutralizing antibodies after SARS-CoV-2 infection or vaccination (ClinicalTrials.gov Identifier: NCT05315583). This study was approved by lie-de-France IV ethical committee.<br>The "Lyon" cohort is an ongoing prospective, multicentric, longitudinal, interventional cohort clinical study (COVID-SER) conducted at Hospices Civils de Lyon. The objective is to evaluate the effectiveness of commercially developed serological test kits currently in development, which will be used for the diagnosis of patients with suspected SARS-CoV-2 infection. A sub-study aimed to build a collection of biological samples and transfer of residual blood products to external partners for the advancement of scientific knowledge on SARS-CoV-2 {ClinicalTrials.gov identifier: NCT04341142).

Note that full information on the approval of the study protocol must also be provided in the manuscript.

Field-specific reporting

Please select the one below that is the best fit for your research. If you are not sure, read the appropriate sections before making your selection.

☒ Life sciences ☐ Behavioural & social sciences ☐ Ecological, evolutionary & environmental sciences

For a reference copy of the document with all sections, see nature.com/documents/nr-reporting-summary-flat.pdf

Life sciences study design

All studies must disclose on these points even when the disclosure is negative.

Sample size	Given the explanatory nature of the study aiming at describing a phenomenon whose frequency has not yet been established it was not possible to use statistical methods were used to predetermine sample size. We included maximum of participants per group to allow statistical analysis.
Data exclusions	None
Replication	All experiments were performed and verified in multiple replicates as indicated in figure legends.
Randomization	The experiments were not randomized as we tested all available samples. Individuals were included without selection other than those imposed by the entry criteria.
Blinding	For convenience experiments were not blinded. However, the clinical sampling and biological measurements were performed by different teams. Only the final assembly of the data revealed the global view of the results.

Reporting for specific materials, systems and methods

We require information from authors about some types of materials, experimental systems and methods used in many studies. Here, indicate whether each material, system or method listed is relevant to your study. If you are not sure if a list item applies to your research, read the appropriate section before selecting a response.

Materials & experimental systems

n/a	Involved in the study	
	x	Antibodies
	x	Eukaryotic cell lines
x		Palaeontology and archaeology
x		Animals and other organisms
	x	Clinical data
x		Dual use research of concern
x		Plants

Methods

n/a	Involved in the study	
x		ChIP-seq
	x	Flow cytometry
x		MRI-based neuroimaging

Antibodies

Antibodies used

NCP-1 is produced by and Nathalie Morel (CEA). Casirivimab (REGN10933; Regeneron), Imdevimab (REGN10987; Regeneron), Cilgavimab (AZD1061; Astrazeneca), Tixagevimab (AZD8895; AstraZeneca) and Sotrovimab (VIR-7831; GSK) were kind gifts of Thierry Prazuck and Laurent Hocqueloux.
- nucleoprotein (N) antibody NCP-1 (0.1 µg/mL) (ref 35)
- anti-IgG Alexa Fluor 488 (dilution 1:500, Invitrogen; cat# A11029)
- Streptavidin Alexa Fluor 647 (dilution 1:500, Invitrogen; cat# S32357)
- anti-alpha tubulin (dilution 1:100, 66031-1-Ig; Proteintech),
- rabbit anti-cleaved caspase-3 (dilution 1:100, D175; Cell Signaling Technology)
- Phalloidin-Atto 565/633 (dilution 1:500, 75784-1MG-F; Sigma)
- anti-TMPRSS2 VHH-A01-Fc 58 (1 µg/ml) (ref. 57)
- anti-ACE2 VHH-B07-Fc (0.5 µg/ml) (kind gift of A. Brelot)

Validation

The reactivity of NCP-1 to SARS-CoV-2 Nucleocapsid was validated using ELISA binding assays (ref 35). The reactivity of Casirivimab, Imdevimab, Cilgavimab, Tixagevimab and Sotrovimab to the SARS-CoV-2 spike was validated by measuring their neutralizing activity against SARS-CoV-2 in O. Schwartz laboratory. Validation of goat anti-human IgGs has been performed by their providers (Jackson ImmunoResearch and Invitrogen).

Eukaryotic cell lines

Policy information about cell lines and Sex and Gender in Research

Cell line source(s)

IGROV-1 cells were from the NCI-60 cell line panel and have been authenticated (ref 67). Vero E6 and Vero-TMPRSS2 were described previously. 293T (CRL-3216) and U2OS (Cat# HTB-96) cells were obtained from ATCC. U2OS-GFP1-10 and 11 (S-Fuse cells) were derived from U2OS. Vero E6 TMP-2 cells were kindly provided by Dr Makoto Takeda lab.

Authentication

IGROV-1, 293T, U2OS and Vero-derived cells have been authenticated by genotyping (Eurofins).

Mycoplasma contamination

All cells are negative for mycoplasma contamination. Tests are performed every Monday.

Commonly misidentified lines
(See ICLAC register)

None

Clinical data

Policy information about clinical studies
All manuscripts should comply with the ICMJEguidelines for publication of clinical research and a completedCONSORT checklist must be included with all submissions.

Clinical trial registration

NCT04750720 and NCT04341142

Study protocol

Protocols can be accessed on clinicaltrial.gov

Data collection

The Orléans cohort started on August 2020 in Orléans Hospital (Centre hospitalier Réginal Orléans), and is on-going.
The Lyon cohort started on April 2020 at Hospices Civils de Lyon and is on-going.

Outcomes

The primary outcome of the study were the presence of neutralizing antibodies (S-Fuse assay).

Plants

Seed stocks	*Report on the source of all seed stocks or other plant material used. If applicable, state the seed stock centre and catalogue number. If plant specimens were collected from the field, describe the collection location, date and sampling procedures.*
Novel plant genotypes	*Describe the methods by which all novel plant genotypes were produced. This includes those generated by transgenic approaches, gene editing, chemical/radiation-based mutagenesis and hybridization. For transgenic lines, describe the transformation method, the number of independent lines analyzed and the generation upon which experiments were performed. For gene-edited lines, describe the editor used, the endogenous sequence targeted for editing, the targeting guide RNA sequence (if applicable) and how the editor was applied.*
Authentication	*Describe any authentication procedures for each seed stock used or novel genotype generated. Describe any experiments used to assess the effect of a mutation and, where applicable, how potential secondary effects (e.g. second site T-DNA insertions, mosiacism, off-target gene editing) were examined.*

Flow Cytometry

Plots

Confirm that:

- [x] The axis labels state the marker and fluorochrome used (e.g. CD4-FITC).
- [x] The axis scales are clearly visible. Include numbers along axes only for bottom left plot of group (a 'group' is an analysis of identical markers).
- [x] All plots are contour plots with outliers or pseudocolor plots.
- [x] A numerical value for number of cells or percentage (with statistics) is provided.

Methodology

Sample preparation	Cells were stained as indicated in the method section. All samples were acquired within 24h.
Instrument	Attune NxT Acoustic Focusing Cytometer, blue/red/violet/yellow (catalog number : 15360667)
Software	AttuneNxT Software v3.2.1
Cell population abundance	At least 10,000 cells were acquired for each condition.
Gating strategy	All gates were set on unstained cells.

- [x] Tick this box to confirm that a figure exemplifying the gating strategy is provided in the Supplementary Information.
